# Ataxia telangiectasia: Family management

**DOI:** 10.4103/0971-6866.64940

**Published:** 2010

**Authors:** Arun Seshachalam, Sanju Cyriac, Neelesh Reddy, Sagar T. Gnana

**Affiliations:** Department of Medical Oncology, Cancer Institute (WIA), Chennai, India

**Keywords:** Ataxia Telangiectasia, family management, malignancy

## Abstract

Ataxia telangiectasia (AT) is a rare autosomal recessive disease resulting in progressive degeneration of multiple systems in the body. Both A-T homozygote and heterozygote are at increased risk of developing malignancy. We report a family in which three generations were affected by this disorder. Our index case is a 12-year-old female child, born of second degree consanguineous marriage diagnosed to have ataxia telangiectasia at the age of four years, now presented with fever and neck swelling of one month duration. Family history suggestive of ataxia telangiectasia in maternal uncle and younger sibling was present. History of premature coronary artery disease and death in paternal grandfather was present. On evaluation, child was diagnosed to have Alk negative anaplastic large T cell lymphoma. Management included genetic counseling, examination of all the family members, identification of A-T homozygote and providing appropriate care, regular surveillance of the heterozygote for malignancy.

## Introduction

Ataxia-telangiectasia is multisystem, complex disorder first described by Syllaba and Henner in 1926.[1] The syndrome subsequently received the name of Louis Bar, who first described progressive cerebellar ataxia and cutaneous telangiectasia in a Belgian child. The incidence is about 1 in 100,000 live birth. Males and females are equally affected and there are no racial or regional preferences.

## Case Report

The index case is a 12-year-old female child with history of delayed walking and convulsions from 4 years of age, now presenting with progressive neck swelling and fever of one month duration. History of recurrent respiratory tract infection requiring hospital admission is present. Further history reveals that her youngest brother and maternal uncle had similar illness. Her maternal uncle had convulsions and was wheel chair bound at the age of 12 years. He died at 35 years of age because of respiratory tract infection. Paternal grandfather died of myocardial infarction at 45 years of age. There is a healthy second brother among the three siblings. Parents were healthy with no motor disability. There was no unexplained neonatal death, miscarriage or abortion in the family. They were born to second degree consanguineous parents and were full term, normal at birth.

Course of illness started with unsteady gait and easy fall at 4 years of age. Difficulty in walking progressively increased and presently the child is chair bound. These were associated with difficulty in swallowing and speech. History of recurrent sino-pulmonary infections requiring hospital admissions was present. Youngest sibling is 4 years of age and had first episode of convulsion at one and a half years of age. Mother noticed difficulty in walking at 2 years of age and presently he walks with support and has speaking difficulty.

**Figure 1:**
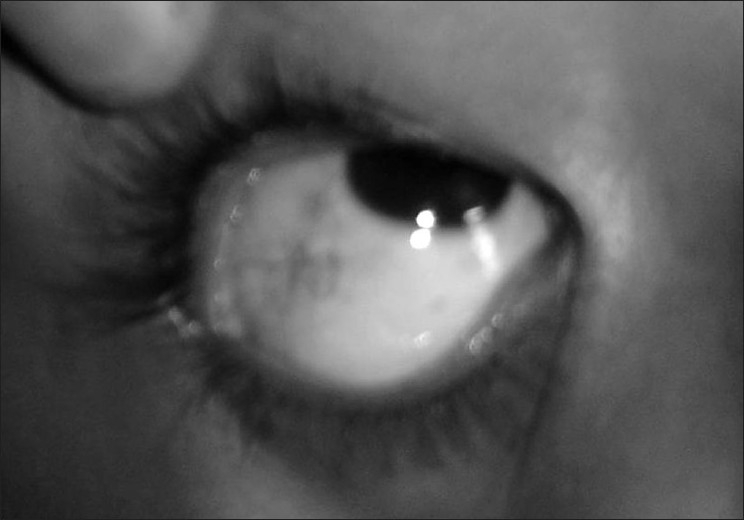
Picture showing telangiectasia over bulbar conjunctivae.

Both the affected siblings were under nourished with growth parameters including head circumference under third percentile. There were no facial dysmorphism, but they have an expressionless face. Both the affected siblings have telangiectasia over bulbar conjunctivae [Figure 1] and the eldest sibling has grey hairs. Bilateral cervical and axilliary lymph node enlargement largest of 3 × 5 cm was present in the eldest sibling.

Cardiovascular, respiratory and abdominal examinations were within normal limits. Walking was impossible without support in both the cases, pes cavus is noticed in the index case. Significant mental slowness with poor response was present. Features of cerebellar dysfunction such as cerebellar ataxia, dys-synergia, dys-arthia and intentional tremors are present. Deep tendon reflexes are diminished and the plantar responses are flexor. Ocular apraxia could also be demonstrated.

### Investigation

**Figure 2:**
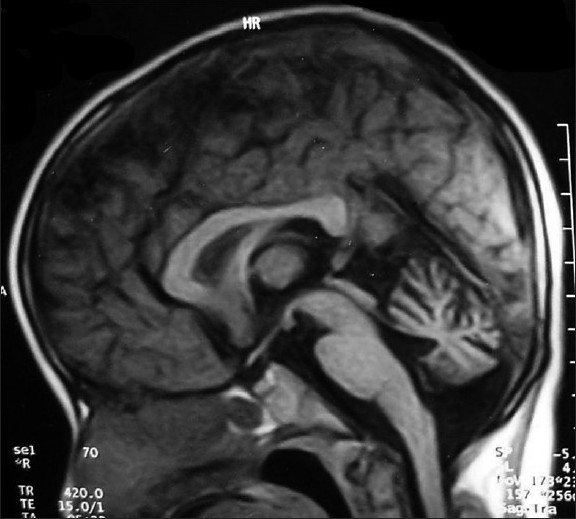
MRI Brain showing cerebellar atrophy.

AFP and CEA, which are markers of A-T, were checked. Both the affected sibling have raised AFP and normal CEA level. Dys-gamaglobinemia and MRI Brain showing cerebellar atrophy is found in the elder sibling [Figure 2].

Biopsy of axillary node in elder sibling was suggestive of anaplastic large cell lymphoma (CD15-, CD30+, ALK+).

## Discussion

Ataxia-telangiectasia (AT) is an autosomal recessive disorder that has multisystem manifestations including motor impairments secondary to a neurodegenerative process, oculocutaneous telangiectasia, progressive immunodeficiency, chronic sinopulmonary infections, increased risk of lympho-reticular cancer, and hypersensitivity to ionizing radiation. The disease is heterogeneous, both clinically and genetically, as shown by the existence of four complementation groups (A, C, D, E). The responsible gene (*ATM* gene) has been mapped to band11q22-23.

AT is a rare disease with a prevalence estimated to be less than 1 in 100 000.[2–4] The frequency of A-T mutant allele heterozygosity was reported to be 1.4-2% of the general population. Death typically occurs in early or middle adolescence, usually from bronchopulmonary infection, less frequently from malignancy, or from a combination of both. The median age at death is reported to be approximately 20. The lifetime risk of cancer among patients with A-T has been estimated to be 10-38% which is about 100-fold more than the population rate; however, in the absence of chronic bronchopulmonary disease and lymphoreticular malignancy, A-T is consistent with survival into the fifth or sixth decade.

A-T heterozygotes present an excess risk of death (they die 7-8 y earlier than the normal population), mostly from ischemic heart disease (A-T carriers die 11 y younger than noncarriers) or cancer (A-T carriers die 4 y younger than noncarriers).[5–7] It was estimated that up to 8% of all cases of breast cancer are AT carriers. They are also found to be hypersensitive to ionizing radiation and radiomimetic drugs, thus they may not tolerate certain antineoplastic regimens. Identification of such heterozygote in cancer patients before treatment can allow the use of more appropriate therapeutic regimens.

*Ataxia:* Ataxia has its onset in infancy, becoming apparent when the child begins to walk and is relentlessly progressive. From this early stage, ataxia is associated with abnormal head movements and is slowly and steadily progressive; however, the normal development of motor skills between ages 2 and 5 years tends to mask the progression of ataxia, so that parents may report an actual improvement in gait. At this point, a diagnosis of cerebral palsy, ataxic or athetoid is frequently made, but children who are affected have a peculiar gait like little clowns; this finding is highly suggestive of A-T.

*Telangiectasia:* These are dilated vessels usually found at corners of eyes, or on the surface of the ears and cheeks exposed to sunlight. They are noticed after age 3-6 years and sometimes not until adolescence. The mechanism of developing telangiectasia is still unknown.

*Immunodeficiency:* It affects approximately 70% of AT patients. Deficient levels of IgA and IgE are found and render them prone to sinopulmonary infections.

*Predisposition to Cancer:* Cancer is 1, 00 times more frequent in AT than in the general population. Lymphoma and leukemia are particularly common. A-T heterozygote is at increased risk of breast cancer. It is fascinating that these patients tend to have elevated AFP and CEA, both of which are tumor markers. These two substances are produced normally during fetal development, but the production is inhibited after birth. It is possible that this inhibition is one of the roles of the ATM protein. Some interesting questions would be whether the levels of these markers relate to their proneness to cancer, and whether changes in their levels can be used for early cancer detection during follow-up. Further large-scale studies would be needed to answer these questions.

### Other Possible Symptoms

- Mask face
- Absence or dysplasia of thymus gland
- Choreoathetosis
- Dystonia
- Slowed growth
- Proneness to insulin-resistant diabetes in adolescence
- Progeric changes in hair and skin and progeric vascular changes

Diagnostic Criteria isformulated by Ataxia-Telangiectasia Clinical Center at the Johns Hopkins Medical Institutions, which is the following:[8]

Ataxia or significant motor incoordination with raised alpha fetoprotein (AFP) (>2x) +3 of the following four *characteristic clinical features:*

1. Incoordination of head and eyes in lateral gaze deflection
2. Ocular telangiectasia
3. Gait ataxia associated with an inappropriately narrow-base
4. Immunoglobulin deficiencies

Patients with less than three of these characteristics were required to have the diagnosis confirmed by the finding of radiation-induced chromosomal breaks in lymphocytes. Siblings of known patients with AT who are older than 1 year of age and had ataxia only needed to have an elevated AFP.

Both the siblings, who fulfilled the above criteria and were diagnosed as ataxia telangiectasia index case, were evaluated for neck swelling and diagnosed to have anaplastic large cell lymphoma – stage II b. In view of progressive neurological disease and adverse effects of chemotherapeutic agents, parents declined chemotherapy and opted for supportive management. The mother of these two siblings is an obligate carrier and is known to have increased risk of breast cancer. However, the benefit of frequent screening mammograms must be balanced against the risk of radiosensitivity. The current opinion is to have the routine screening for breast cancer. The carrier status of the unaffected brother is estimated to be 50%, but definitive test to identify this would be very helpful in counseling. All the other family members were thoroughly examined and genetic counseling was provided to them. They were also educated regarding the nature of the disease and the need to have regular screening for malignancy.

## Conclusion

Ataxia telangiectasia is mainly a clinical diagnosis and unnecessary, costly investigations should be avoided. Management includes genetic counseling, examination of all the family members, identification of A-T homozygote and providing appropriate care, regular surveillance of the heterozygote for malignancy.
